# The Effect of Bilateral Nephrectomy on Renalase and Catecholamines in Hemodialysis Patients

**DOI:** 10.3390/ijerph18126282

**Published:** 2021-06-10

**Authors:** Magda Wiśniewska, Natalia Serwin, Violetta Dziedziejko, Małgorzata Marchelek-Myśliwiec, Barbara Dołęgowska, Leszek Domański, Kazimierz Ciechanowski, Krzysztof Safranow, Tomasz Gołębiowski, Andrzej Pawlik

**Affiliations:** 1 Clinical Department of Nephrology, Transplantology and Internal Medicine, Pomeranian Medical University, 70-111 Szczecin, Poland; mwisniewska35@gmail.com (M.W.); malgorzata.marchelek@gmail.com (M.M.-M.); domanle@pum.edu.pl (L.D.); kazcie@pum.edu.pl (K.C.); 2 Immunology and Laboratory Medicine, Department of Microbiology, Pomeranian Medical University, 70-111 Szczecin, Poland; nat.serwin@gmail.com (N.S.); barbara.dolegowska@pum.edu.pl (B.D.); 3 Department of Biochemistry and Medical Chemistry, Pomeranian Medical University, 70-111 Szczecin, Poland; viola@pum.edu.pl (V.D.); chrissaf@mp.pl (K.S.); 4 Department of Nephrology and Transplantation Medicine, Wroclaw Medical University, 50-556 Wroclaw, Poland; tomasz.golebiowski@umed.wroc.pl; 5 Department of Physiology, Pomeranian Medical University, 70-111 Szczecin, Poland

**Keywords:** renalase, kidney, erythrocytes, bilateral nephrectomy, chronic kidney disease

## Abstract

Background/Aims: Renalase is an enzyme with monoamine oxidase activity that metabolizes catecholamines; therefore, it has a significant influence on arterial blood pressure regulation and the development of cardiovascular diseases. Renalase is mainly produced in the kidneys. Nephrectomy and hemodialysis (HD) may alter the production and metabolism of renalase. The aim of this study was to examine the effect of bilateral nephrectomy on renalase levels in the serum and erythrocytes of hemodialysis patients. Methods: This study included 27 hemodialysis patients post-bilateral nephrectomy, 46 hemodialysis patients without nephrectomy but with chronic kidney disease and anuria and 30 healthy subjects with normal kidney function. Renalase levels in the serum and erythrocytes were measured using an ELISA kit. Results: Serum concentrations of renalase were significantly higher in post-bilateral nephrectomy patients when compared with those of control subjects (101.1 ± 65.5 vs. 19.6 ± 5.0; *p* < 0.01). Additionally, renalase concentrations, calculated per gram of hemoglobin, were significantly higher in patients after bilateral nephrectomy in comparison with those of healthy subjects (994.9 ± 345.5 vs. 697.6 ± 273.4, *p* = 0.015). There were no statistically significant differences in plasma concentrations of noradrenaline or adrenaline. In contrast, the concentration of dopamine was significantly lower in post-nephrectomy patients when compared with those of healthy subjects (116.8 ± 147.7 vs. 440.9 ± 343.2, *p* < 0.01). Conclusions: Increased serum levels of renalase in post-bilateral nephrectomy hemodialysis patients are likely related to production in extra-renal organs as a result of changes in the cardiovascular system and hypertension.

## 1. Introduction

Renalase is a flavoprotein with enzymatic activity that was identified by Xu et al. in 2005 [1]. Renalase is mainly secreted by the proximal tubules of the kidney, as well as the muscles, liver and cardiomyocytes. The expression of renalase has also been detected in the nervous system, endothelium, adrenals and adipose tissue [2]. The initial proposed function of renalase was to metabolize catecholamines; therefore, it has a significant influence on arterial blood pressure regulation and the development of cardiovascular diseases [3,4]. Recent studies have shown that the apparent catalytic function of renalase is to oxidize isomeric forms of b-NAD(P)H that are reduced at the 2 or 6 positions of the nicotinamide [3,4,5,6]. In addition, renalase exhibits the properties of a cytoprotective cytokine. The cytoprotective properties of renalase are mediated by the receptor PMCA4b, which is a plasma membrane calcium ATPase that regulates local calcium concentrations [7,8]. Its N-terminal peptide, by binding to the calcium-transporting ATPase PMCA4b, activates the PI3K/AKT and MAPK pathways. In patients with chronic kidney disease, renalase could reduce the expression of mediators involved in the inflammatory response and glomerular fibrosis, such as pro-inflammatory cytokines and NADPH oxidase [9]. Recombinant renalase diminishes glomerular hypertrophy, interstitial fibrosis, proteinuria, hypertension and myocardial remodeling by decreasing the phosphorylation of ERK-1/2 [9]. It has been shown that renalase may also protect against cisplatin and hydrogen peroxide-induced toxic injury of proximal tubular (HK-2) cells [8,10]. Renalase increases the expression of extracellular signal-regulated kinase (ERK), protein kinase B and p38 mitogen-activated kinase, activating a receptor-mediated pro-survival signaling cascade [11]. Renalase also decreases oxidative stress and has renoprotective properties. In rats with contrast-induced nephropathy, renalase inhibited the development of apoptosis, inflammation and tubular necrosis [12]. Previous studies have shown that renalase is involved in catecholamine metabolism [1,2]. A renalase-deficient mouse displayed increased plasma levels of epinephrine, norepinephrine and dopamine in comparison with those of wild-type mice. Recombinant renalase in renalase-deficient mice caused a significant decrease in plasma levels of epinephrine, norepinephrine and dopamine [13].

Renalase synthesis is regulated by catecholamines [14,15], and previous studies have shown increased serum levels of renalase in patients with end-stage renal disease [16]. Renalase has also been investigated in patients with diabetes, hypertension, heart failure, coronary artery disease and other cardiovascular diseases [17,18,19,20,21]. In these studies, serum renalase was found to be correlated with disease progression as well as cardiovascular complications. Although renalase is mainly secreted by the kidneys, this enzyme may be produced by other tissues, such as cardiomyocytes and myocytes. Nephrectomy and hemodialysis (HD) treatment may alter the production and metabolism of renalase [22,23]. In our previous studies, we examined renalase concentrations in patients with chronic kidney diseases with residual kidney function and on hemodialysis [24,25]. Our results indicated that renalse levels were increased in serum and decreased in erythrocytes. In addition, renalase concentrations were correlated with impaired kidney function.

The physiological role of renalase, its involvement in the regulation of arterial pressure and organ blood flow, as well as its effects on renal function remain incompletely understood. The better understanding of the role of renalase under normal physiological conditions requires further studies. 

The aim of this study was to examine the renalase and catecholamines in the serum and erythrocytes of hemodialysis patients after bilateral nephrectomy. To our knowledge, a concentration assessment of renalase in erythrocytes in patients after bilateral nephrectomy has never been performed. 

## 2. Material and Methods

### 2.1. Patients

This study included the following participants: 27 Caucasian hemodialysis patients (15 males and 12 females, mean age 52.5 ± 13.5 years, mean dialysis duration 769 ± 547 days) who were post-bilateral nephrectomy; 46 hemodialysis patients without nephrectomy, but with chronic kidney disease and anuria (30 males and 16 females), with a mean age of 66.2 ± 16.8 years and mean dialysis duration of 910 ± 729 days; and 30 healthy subjects with normal kidney function (normal GFR values and normal creatinine serum concentrations; (15 males and 15 females) and a mean age of 57.4 ± 18.5 years [24,25]. The causes of bilateral nephrectomy were polycystic kidney disease in 10 patients, glomerular kidney disease in 1 patient, neoplasmatic diseases in 8 patients, birth defects in 4 patients and other causes in 4 patients. Hypertension was diagnosed in 20 (74.1%) patients after bilateral nephrectomy, in 41 (89.1%) hemodialysis patients without nephrectomy, but with chronic kidney disease and anuria, and in 9 (30%) healthy control subjects with normal kidney function. All participants from the study groups were patients of the Clinic of Nephrology, Transplantology and Internal Diseases, Pomeranian Medical University in Szczecin. The ethics committee of the Pomeranian Medical University in Szczecin, Poland approved the study (KB-0012/122/14). 

### 2.2. Methods

Renalase concentrations in serum and erythrocytes were measured using an ELISA kit specific for human renalase (WuHan EIAab, Wuhan, China). Basic biochemical parameters were measured using commercially available reagent kits (BioMaxima, Lublin, Poland). Plasma concentrations of adrenaline, noradrenaline and dopamine were measured using an ELISA kit (LDN Labor Diagnostika Nord GmbH & Co. KG, Nordhorn, Germany) [24]. In the group of HD patients, blood was sampled immediately prior to HD. 

For serum samples, blood was drawn into Sarstedt S-Monovette tubes with a clotting activator, left for 30 min at room temperature, centrifuged (1000× *g*, 10 min, room temperature), distributed into fresh vials and kept frozen at −80 °C until assays were performed. Erythrocytes were obtained from whole blood collected in S-Monovette tubes with a K3EDTA anticoagulant, and then mixed thoroughly and centrifuged (1000× *g*, 10 min, room temperature). Next, blood plasma was removed from the tubes, placed in fresh vials and frozen until further use in catecholamine assays. Erythrocytes remaining in the tube were washed three times by adding saline (0.9% NaCl), mixing thoroughly, centrifuging as mentioned above and then removing the saline. Following the third wash, the saline was removed from the tubes, and the erythrocyte suspensions were distributed to fresh vials and then frozen.

Prior to performing the immunoenzymatic assays, the erythrocyte suspensions were thawed, diluted 1:3 with ultrapure water (50 µL of erythrocytes + 150 µL of ultrapure water) and vortexed intensely for 2 min. The obtained hemolysates were analyzed for hemoglobin concentration using Drabkin’s method: 20 µL of obtained hemolysates were added to 1 mL of Drabkin’s reagent, which was vortexed and incubated for 30 min at room temperature. Then, the extinction of analyzed samples was measured spectrophotometrically at 540 nm with 1 mL of Drabkin’s reagent serving as the blank and 1 mL of cyanmethemoglobin serving as the standard. The concentration of hemoglobin was calculated using the following formula: Hb [g/100 mL] = EB/Es × cS × 0.0502, where EB is the extinction of the particular sample, ES is the extinction of the standard, cS is the standard concentration and 0.0502 is the coefficient resulting from the dilution.

The same hemolysates (diluted 1:3 in the first step) were used directly in the immunoenzymatic assays according to the manufacturer’s instructions. If the hemolysates were too concentrated (more than 5 g/dL of hemoglobin in the lysate), the samples were further diluted with ultrapure water to obtain hemoglobin concentrations in the range of 4–5 g/dL. The total concentration of renalase in the erythrocytes (expressed in ng/mL), as well as the concentration of renalase expressed per gram of Hb (ng/g Hb), are presented. Renalase concentration was expressed per gram of Hb to account for variability in the patients’ erythrocyte and hemoglobin values. 

### 2.3. Statistical Analysis

The normality of the distributions of the studied parameters was verified using the Shapiro–Wilk test. Because of a significant deviation from normality for the majority of the quantitative variables, a non-parametric Mann–Whitney U test was used to compare values between groups. Correlations between selected quantitative parameters were investigated using Spearman’s rank correlation coefficient. Values of quantitative variables were reported as mean and standard deviation (±SD). Differences were considered statistically significant at *p* < 0.05. A sample size equal to 27, 46 and 30 subjects in the after bilateral nephrectomy, on hemodialysis without nephrectomy and healthy control groups, respectively, was sufficient to provide 80% statistical power at the 0.05 significance level for the detection of an effect size corresponding to differences between the groups equal to 0.69 SD for bilateral nephrectomy vs. hemodialysis, 0.76 SD for bilateral nephrectomy vs. healthy controls and 0.67 SD for hemodialysis without nephrectomy vs. healthy controls. Data were analyzed using STASTISTICA 12.5 software (StatSoft, Tulsa, OK, USA). 

## 3. Results 

**Figure 1:**
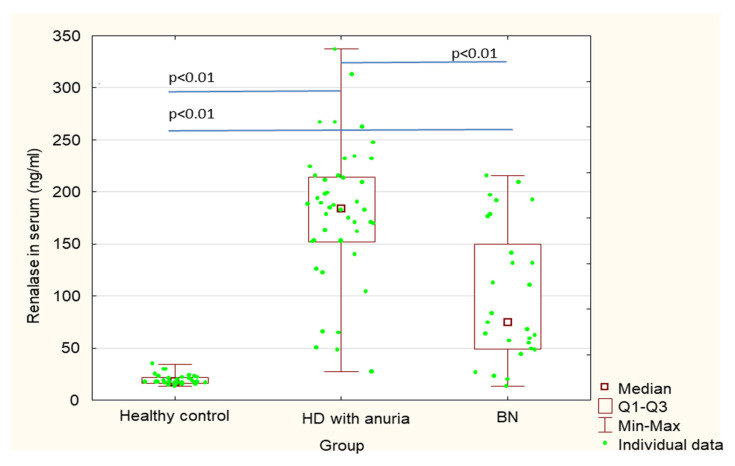
Serum concentrations of renalase in patients after bilateral nephrectomy, control healthy subjects and hemodialysis patients without nephrectomy, but with chronic kidney disease and anuria.

**Figure 2:**
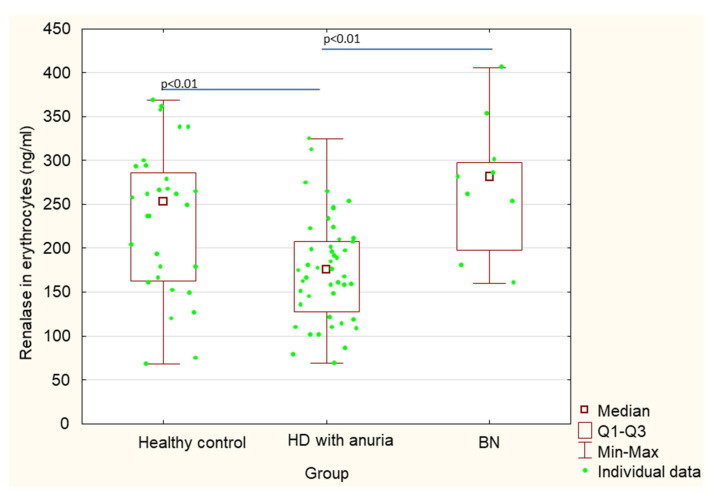
Erythrocyte concentrations of renalase in patients after bilateral nephrectomy, control healthy subjects and hemodialysis patients without nephrectomy, but with chronic kidney disease and anuria.

**Figure 3:**
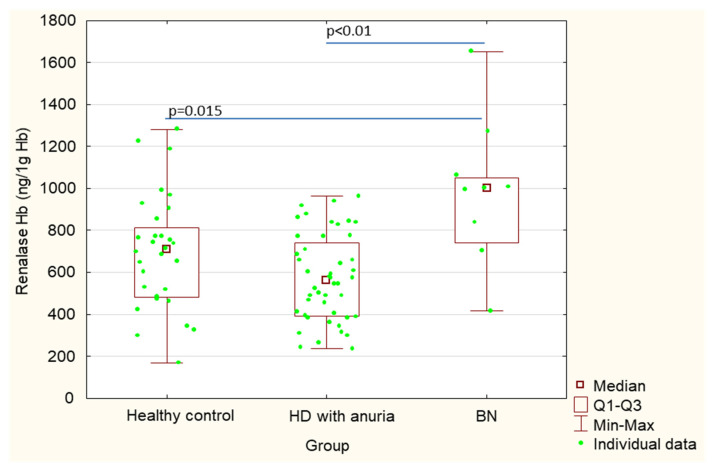
Renalase concentrations calculated per hemoglobin (ng of renalase/g hemoglobin) in lysates of erythrocytes in patients after bilateral nephrectomy, control healthy subjects and hemodialysis patients without nephrectomy, but with chronic kidney disease and anuria.

Table 1 shows a comparison of patients after bilateral nephrectomy, control healthy subjects and hemodialysis patients without nephrectomy, but with chronic kidney disease and anuria. Serum renalase values in post-nephrectomy patients were significantly higher in comparison with control subjects (101.1 ± 65.5 vs. 19.6 ± 5.0; *p* < 0.01) (Table 1, Figure 1). There were no statistically significant differences in erythrocyte absolute renalase concentrations between post-nephrectomy patients and healthy control subjects (275.8 ± 76.7 vs. 233.2 ± 83.1 *p* = 0.18); although, renalase concentrations calculated per gram of hemoglobin (renalase ng/g Hb) were significantly higher in the post-bilateral nephrectomy patients in comparison with healthy subjects (994.9 ± 345.5 vs. 697.6 ± 273.4, *p* = 0.015) (Table 1, Figure 2 and Figure 3).

Additionally, we compared renalase concentrations between patients after bilateral nephrectomy and chronic kidney disease hemodialysis patients with anuria. Serum renalase concentrations in patients after bilateral nephrectomy were significantly lower in comparison with those in hemodialysis patients with anuria (101.1 ± 65.5 vs. 177.2 ± 68.3, *p* < 0.01) (Table 1, Figure 1). In contrast, renalase concentrations in erythrocytes were significantly higher in patients after bilateral nephrectomy in comparison with those in hemodialysis patients with anuria (275.8 ± 76.7 vs. 176.4 ± 59.1, *p* < 0.01). Similarly, renalase concentrations calculated per gram of hemoglobin (renalase ng/g Hb) were significantly higher in patients after bilateral nephrectomy in comparison with those in hemodialysis patients with anuria (994.9 ± 345.5 vs. 573.1 ± 205.8, *p* < 0.01 (Table 1, Figure 2 and Figure 3).

Serum renalase concentrations in hemodialysis patients with chronic kidney disease were significantly higher than those in healthy control subjects (177.2 ± 68.3 vs. 19.6 ± 5.0, *p* < 0.01), whereas renalase concentrations in erythrocytes were significantly lower (176.4 ± 59.1 vs. 233.2 ± 83.1, *p* < 0.01) (Table 1, Figure 1 and Figure 2).

There were no statistically significant differences in plasma concentrations of adrenaline and noradrenaline between patients after bilateral nephrectomy and healthy control subjects, whereas plasma concentrations of adrenaline were significantly higher in patients after bilateral nephrectomy than in hemodialysis patients with chronic kidney disease (65.4 ± 129.1 vs. 15.3 ± 12.9, *p* < 0.01). Meanwhile, the post-nephrectomy group demonstrated lower plasma concentrations of dopamine in comparison with healthy subjects and hemodialysis patients with chronic kidney disease (116.8 ± 147.7 vs. 440.9 ± 343.2, vs. 179.0 ± 125.2, *p* < 0.01 for both).

Table 2 summarizes the correlations between renalase and the other measured parameters, which included serum and erythrocyte renalase, renalase concentrations calculated per gram of hemoglobin (Renalase Hb), plasma concentrations of adrenaline, noradrenaline and dopamine, duration of hemodialysis, glucose, uric acid, total protein in serum, albumin, hemoglobin, mean concentration of hemoglobin in erythrocytes and erythrocyte number. These correlations were not statistically significant, except for those positive correlations between the absolute concentration of renalase in erythrocytes, renalase concentrations calculated per gram of hemoglobin and serum albumin. Renalase in erythrocytes calculated per gram of hemoglobin was positively correlated with the mean concentration of hemoglobin in erythrocytes.

In addition, renalase levels were compared between patients with normal blood pressure values and patients with hypertension in the three study groups. In patients after bilateral nephrectomy and patients with CKD, there were no statistically significant differences in renalase concentrations between patients with normal blood pressure values and patients with hypertension. Among subjects with normal renal function, statistically significantly increased serum renalase levels were observed in hypertensive subjects. Erythrocyte renalase concentrations were also increased in hypertensive subjects. However, this difference had borderline statistical significance (*p* = 0.07) (Table 3). 

## 4. Discussion

The aim of this study was to examine the concentrations of renalase in the serum and erythrocytes of hemodialysis patients after bilateral nephrectomy. In our previous studies, renalase was investigated in patients with chronic kidney disease with preserved renal function who did not require hemodialysis and in patients with chronic kidney disease on hemodialysis. In the present study we examined renalase in patients after bilateral nephrectomy. So far, only a few studies have examined renalase in patients after nephrectomy. To our knowledge, this constitutes the first study examining the concentrations of renalase in erythrocytes in these patients [24,25].

The serum concentrations of renalase were significantly higher in post-bilateral nephrectomy hemodialysis patients in comparison with those of healthy subjects, but they were lower than those in hemodialysis patients with chronic kidney disease and anuria. Our results suggest that other organs, in addition to the kidneys, may serve as sources of serum renalase.

Additionally, we examined the renalase concentrations in erythrocytes. Our results indicated that the renalase concentrations in erythrocytes in patients after bilateral nephrectomy were significantly higher than those in hemodialysis patients with chronic kidney disease, whereas renalase concentrations, calculated per gram of hemoglobin, were significantly higher in patients after bilateral nephrectomy than those in healthy controls and hemodialysis patients with chronic kidney disease. The concentration of renalase in erythrocytes calculated per gram of hemoglobin is the most reliable parameter to describe the concentration of renalase in erythrocytes due to variations in erythrocyte and hemoglobin values between patients.

Patients with CKD have high levels of anemia, which is mainly associated with impaired erythropoietin production, increased hemolysis and significantly shortened erythrocyte survival [26,27]. Hemodialysis can enhance these processes. Previous studies have shown that erythrocytes as nuclear-free cells do not carry out classical metabolism and are good “transporters” of many substances, including enzymes and cytokines [28]. Renalase could also be subject to such transport. Moreover, the renalase receptor PMCA4b was found in erythrocytes [7]. Renalase is also responsible for autocrine functions and exhibits enzymatic activity mainly at the intracellular level, stabilizing the active (NAD(P)H) isoform [29]. There are many types of enzymes in erythrocytes, mostly antioxidants requiring NAD(P)H [29]. Our results indicate that renalase may be stored and transported in erythrocytes, which may also be the source of serum renalase.

To date, post-nephrectomy renalase levels have not been studied extensively. Taranta-Janusz et al. assessed serum and urine renalase levels in children with a single kidney [30]. The authors showed that renalase levels in patients after unilateral nephrectomy were significantly lower in comparison with those of healthy subjects and decreased with disease progression [30]. Yin et al. examined the role of renalase in the progression of cardiorenal syndrome (CRS) after subtotal nephrectomy in an animal model [31]. These authors found decreased cardiac remodeling, diminished hypertrophy of cardiomyocytes and cardiac interstitial fibrosis and lower blood pressure in rats treated with renalase. Moreover, the authors demonstrated that renalase inhibits the expression of extracellular signal-regulated kinase-1/2, an enzyme involved in oxidative stress and inflammation [31].

Similar results were obtained by Baraka et al., who examined whether renalase administration might decrease the severity of cardiovascular disease in rats after subtotal nephrectomy [32]. The authors observed the amelioration of cardiovascular parameters in rats after the administration of renalase. Malyszko et al. examined serum renalase concentrations in subjects with chronic kidney disease after unilateral or bilateral nephrectomy on hemodialysis or hemodiafiltration, as well as the levels in the urine and ultrafiltrate of hemodialysis subjects [33]. Renalase concentrations in the urine were higher in the control group compared with those in HD subjects. The anuric group had higher renalase concentrations compared with those with remaining diuresis. In patients with residual diuresis, renalase concentrations in the plasma correlated with concentrations in the urine. Moreover, urinary renalase correlated with residual diuresis. Patients on hemodiafiltration had significantly lower renalase levels than did hemodialysis patients.

Several studies have assessed the associations between kidney function and renalase. Xu et al. demonstrated significantly lower plasma concentrations of renalase in patients with end-stage renal disease compared with those of controls [1], results that are contrary to ours. However, Stojanovic et al. observed renalase values that correlated positively with serum creatinine and negatively with eGFR [34]. Likewise, Zbroch et al. showed increased serum renalase concentrations in kidney transplant recipients [35]. They found that renalase correlated positively with serum creatinine, systolic and diastolic blood pressure, age and time since transplantation. Additionally, Przybylowski et al. showed increased levels of renalase in heart transplant recipients, which were correlated with kidney function [36,37]. Increased renalase levels were observed in patients with lower e-GFR and impaired kidney function. The above studies suggest that chronic kidney disease and impaired kidney function are associated with increase in renalase levels, possibly through a compensatory mechanism. Regardless of the cause and severity, chronic kidney disease alone is sufficient to stimulate the adrenergic system to increase renal renalase synthesis due to a low level of inflammation, activation of oxidative stress and the renin-angiotensin-aldosterone system. In our study, the serum levels of epinephrine and norepinephrine in patients after nephrectomy were higher than those in the healthy control group, although this difference was not statistically significant. We suspect that this was probably related to a low sample size. Results of our earlier work including 155 participants showed a positive relationship between the levels of those two catecholamines and the chronic kidney disease stage [24]. It should also be taken into account that nephrectomy may itself be a factor stimulating renalase activation. Data from animal studies have indicated that in rats subjected to renal denervation, the renalase concentration increased significantly in the first week and then decreased but remained at a higher level after 6 weeks [38].

The cause of the above contradictory results has not been precisely established. It is suspected that the use of various tests may be an important contributing factor. In our study, a commercial ELISA kit was used to assess both serum and erythrocyte renalase; therefore, our results are consistent with those of other authors using this method. The advantage of the immunoenzymatic ELISA method compared with Western blot analysis is that it is a quantitative method, whereas Western blot is a semi-quantitative method. The ELISA method used in the evaluation of serum renalase concentrations seems to be more suitable for such analyses.

To our surprise, the concentration of renalase in the serum did not correlate with that in erythrocytes. We are not able to provide the reason for this phenomenon. Perhaps it is related to the individual characteristics of patients, who may differ in the number of renalase receptors on their erythrocytes or in their intracellular renalase metabolism. It may also result from renalase production by other, non-renal organs. In our previous study in non-dialyzed chronic kidney disease patients, we showed that, with the advancement of chronic kidney disease, the concentration of renalase in the erythrocyte lysate increased progressively [24]. At present, the renalase concentration was significantly higher in patients after nephrectomy than in the control group. We believe that this may indicate a role for erythrocytes in the transport of renalase. This issue will require further research on the role of erythrocytes in renalase function and its significance in physiology and uremia.

In the present study, we observed increased serum renalase levels in patients after bilateral nephrectomy in comparison with those of healthy subjects, whereas these concentrations were lower than those in hemodialysis patients with chronic kidney disease. Moreover, renalase concentrations in erythrocytes and those calculated per gram of hemoglobin were significantly higher in patients after bilateral nephrectomy than those in healthy controls and in hemodialysis patients with anuria. We compared renalase levels between hemodialysis patients after bilateral nephrectomy and hemodialysis patients without nephrectomy, with chronic kidney disease and anuria; therefore, we can exclude renalase elimination in the urine. Previous studies have shown that renalase synthesis is primarily impaired in patients with chronic kidney disease and increases with disease progression [1]. We suppose that decreased renalase concentrations in the erythrocytes of hemodialysis patients with chronic kidney disease may be primarily due to decreased renalase production by the kidneys in these patients, whereas increased serum levels may be the result of compensatory renalase synthesis in this disease. Increased serum renalase levels in hemodialysis patients after bilateral nephrectomy and hemodialysis patients with chronic kidney disease may be caused by compensatory production in extra-renal organs as a result of changes in the circulatory system and hypertension. However, we cannot exclude the possibility that elevated serum renalase levels in hemodialysis patients after bilateral nephrectomy and CKD patients with anuria may also be due to a lack of renal excretion of renalase. The results of this study indicate that nephrectomy causes a number of changes leading to the increased synthesis of renalase in extra-renal organs, which is reflected by the increased concentration of renalase in the serum and erythrocytes.

Our results also indicate that the increase in renalase synthesis in hemodialysis patients with chronic kidney disease is mainly expressed in the serum. Moreover, the increase in serum renalase concentrations in hemodialysis patients with chronic kidney disease is greater than that in hemodialysis patients after bilateral nephrectomy. Chronic kidney disease is characterized by a number of metabolic changes and changes in the circulatory system that may increase serum renalase levels more strongly than bilateral nephrectomy.

Previous studies suggest an association between renalase and hypertension, but the results are often inconsistent [17,21,39,40,41,42,43]. The lack of analysis of correlation between renalase concentrations and blood pressure values is a limitation of our study. In our study, we could not reliably correlate renalase concentrations with blood pressure values because of the large variation in blood pressure in hemodialysis patients. This was related to the process of hemodialysis, the varying degrees of hydration of these patients and the diurnal fluctuations in blood pressure. In our study, we compared renalase levels in patients with and without hypertension in the three study groups. Among patients after bilateral nephrectomy and CKD anuric patients, there were no statistically significant differences. Only among subjects with normal kidney function, we observed increased renalase concentrations in hypertensive subjects. Our results suggest that renalase levels may be elevated in hypertensive subjects with normal renal function, whereas in hemodialysis patients after bilateral nephrectomy and CKD, hypertension is not a significant factor affecting renalase levels. In these patients, there are many other factors, such as lack of renal renalase elimination, hemodialysis and metabolic and endocrine disorders, which affect renalase levels.

Our study has several other limitations such as a limited number of patients and incomplete patient characteristics; however, we believe that it provides important information about renalase and catecholamines in patients after bilateral nephrectomy.

## 5. Conclusions

In conclusion, our study demonstrates increased serum renalase concentrations in post-bilateral nephrectomy hemodialysis patients in comparison with subjects with normal kidney function. We suspect that higher serum renalase in patients after bilateral nephrectomy is related to production in extra-renal organs resulting from changes in the cardiovascular system and hypertension in hemodialysis patients. However, the lack of renal elimination of renalase in these patients may also be the factor that increases its serum levels. We suggest that erythrocytes may serve as renalase transporters and, to a lesser extent, as a source of renalase.

## Figures and Tables

**Table 1:** Comparison of studied parameters between patients after bilateral nephrectomy, control healthy subjects and hemodialysis patients without nephrectomy, but with chronic kidney disease and anuria.

Parameters	Patients Post Bilateral Nephrectomy	Healthy Controls	Hemodialysis Patients with Anuria	Patients Post Bilateral Nephrectomy vs. Healthy Controls	Patients Post Bilateral Nephrectomy vs. Hemodialysis Patients with Anuria	Healthy Controls vs. Hemodialysis Patients with Anuria
	Mean ± SD			*p* *		
Age (years)	52.5 ± 13.5	57.4 ± 18.5	66.2 ± 16.8	0.52	<0.01	0.038
Glucose (mg/dL)	98.2 ± 15.6	86.7 ± 7.2	104.1 ± 31.6	<0.01	0.85	<0.01
Creatinine (mg/dL)	7.10 ± 3.80	0.83 ± 0.10	8.51 ± 3.68	<0.01	0.060	<0.01
Uric acid (mg/dL)	5.0 ± 2.8	5.5 ± 0.6	7.1 ± 1.7	0.033	<0.01	<0.01
Total protein in serum (g/L)	7.54 ± 1.07	7.44 ± 0.43	5.70 ± 0.86	0.85	<0.01	<0.01
Albumin (g/L)	3.75 ± 0.63	3.70 ± 0.16	3.16 ± 0.51	0.44	<0.01	<0.01
HGB (mmol/L)	6.3 ± 0.6	7.9 ± 0.6	6.8 ± 1.1	<0.01	0.033	<0.01
RBC (T/L)	3.6 ± 0.4	4.8 ± 0.4	3.6 ± 0.6	<0.01	0.77	<0.01
MCHC (mmol/L)	20.3 ± 0.5	19.5 ± 0.6	20.6 ± 0.6	<0.01	0.023	<0.01
Adrenaline (pg/mL)	65.4 ± 129.1	30.4 ± 33.9	15.3 ± 12.9	0.11	<0.01	0.039
Noradrenaline (pg/mL)	413 0 ± 579.0	399.1 ± 319.0	301.0 ± 498.2	0.22	0.47	<0.01
Dopamine (pg/mL)	116.8 ± 147.7	440.9 ± 343.2	179.0 ± 125.2	<0.01	<0.01	<0.01
Renalase in serum (ng/mL)	101.1 ± 65.5	19.6 ± 5.0	177.2 ± 68.3	<0.01	<0.01	<0.01
Renalase in erythrocytes (ng/mL)	275.8 ± 76.7	233.2 ± 83.1	176.4 ± 59.1	0.18	<0.01	<0.01
Renalase Hb (ng/1 g Hb)	994.9 ± 345.5	697.6 ± 273.4	573.1 ± 205.8	0.015	<0.01	0.060

* Mann–Whitney U test.

**Table 2:** Correlations between the studied parameters in patients after bilateral nephrectomy.

Parameters	Renalase in Serum		Renalase in Erythrocytes		Renalase Hb	
	Rs	*p*	Rs	*p*	Rs	*p*
Renalase in erythrocytes	−0.03	0.93	-	-	-	-
Renalase Hb	−0.02	0.97	0.93	0.0002	-	-
Glucose	−0.02	0.92	−0.54	0.14	−0.46	0.21
Uric acid	−0.31	0.12	−0.38	0.31	−0.37	0.33
Total protein in serum	−0.41	0.03	0.60	0.08	0.50	0.17
Albumin	−0.34	0.084	0.78	0.01	0.65	0.05
HGB	0.11	0.59	0.27	0.49	0.19	0.62
RBC	0.08	0.68	0.30	0.43	0.23	0.55
MCHC	0.15	0.44	0.82	0.006	0.82	0.007
Adrenaline	0.04	0.87	0.13	0.73	−0.02	0.97
Noradrenaline	−0.10	0.62	0.25	0.52	0.17	0.67
Dopamine	−0.01	0.96	0.17	0.67	0.27	0.49
Duration of hemodialysis	−0.18	0.38	−0.32	0.41	−0.37	0.33

R_s_—Spearman rank correlation coefficient, HGB—hemoglobin, RBC—red blood cells, MCHC—mean corpuscular hemoglobin concentration, Renalase Hb—renalase concentrations calculated as hemoglobin (ng of renalase/1 g hemoglobin in lysates).

**Table 3:** Renalase concentrations in patients with and without arterial hypertension.

Parameters	Patients Post-Bilateral Nephrectomy			Healthy Controls			Hemodialysis Patients with Anuria		
	without HA	with HA	*p* *	without HA	with HA	*p* *	without HA	with HA	*p* *
	Mean ± SD			Mean ± SD			Mean ± SD		
Renalase in serum (ng/mL)	109.4 ± 73.0	98.2 ± 61.9	0.91	18.1 ± 3.8	23.3 ± 5.8	0.007	167.9 ± 59.1	178.3 ± 69.8	1.00
Renalase in erythrocytes (ng/mL)	279.0 ± 7.5	273.3 ± 88.1	0.62	217.7 ± 75.0	269.6 ± 94.2	0.07	173.1 ± 63.8	176.8 ± 59.3	0.99
Renalase Hb (ng/1 g Hb)	936.6 ± 366.6	1041.5 ± 363.2	0.63	640.7 ± 240.7	830.5 ± 312.8	0.09	579.3 ± 289.4	572.3 ± 198.2	0.90

* Mann–Whitney U test, HA—arterial hypertension.

## Data Availability

Data sharing not applicable.
